# Classification of Alzheimer’s Disease and Frontotemporal Dementia Using Electroencephalography to Quantify Communication between Electrode Pairs

**DOI:** 10.3390/diagnostics14192189

**Published:** 2024-09-30

**Authors:** Yuan Ma, Jeffrey Keith Spaneas Bland, Tsutomu Fujinami

**Affiliations:** 1 Development Division, FOVE Inc., Tokyo 107-0061, Japan; jeff.bland@fove-inc.com; 2 School of Knowledge Science, Japan Advanced Institute of Science and Technology, Nomi 923-1292, Japan; fuji@jaist.ac.jp

**Keywords:** dementia, Alzheimer’s disease, frontotemporal dementia, electroencephalogram, machine learning, support vector machine

## Abstract

Accurate diagnosis of dementia subtypes is crucial for optimizing treatment planning and enhancing caregiving strategies. To date, the accuracy of classifying Alzheimer’s disease (AD) and frontotemporal dementia (FTD) using electroencephalogram (EEG) data has been lower than that of distinguishing individuals with these diseases from healthy elderly controls (HCs). This limitation has impeded the feasibility of a cost-effective differential diagnosis for the two subtypes in clinical settings. This study addressed this issue by quantifying communication between electrode pairs in EEG data, along with demographic information, as features to train machine learning (support vector machine) models. Our focus was on refining the feature set specifically for AD-FTD classification. Using our initial feature set, we achieved classification accuracies of 76.9% for AD-HC, 90.4% for FTD-HC, and 91.5% for AD-FTD. Notably, feature importance analyses revealed that the features influencing AD-HC classification are unnecessary for distinguishing between AD and FTD. Eliminating these unnecessary features improved the classification accuracy of AD-FTD to 96.6%. We concluded that communication between electrode pairs specifically involved in the neurological pathology of FTD, but not AD, enables highly accurate EEG-based AD-FTD classification.

## 1. Introduction

The differential diagnosis of dementia subtypes, particularly the most prevalent forms, Alzheimer’s disease (AD) and frontotemporal dementia (FTD), is receiving increasing attention [1,2]. AD and FTD have distinct pathologies and, therefore, may require different treatments. For example, lecanemab has been reported to eliminate amyloid accumulation and slow the progression of mild AD [3], but it may not be similarly effective for FTD. On another note, individuals with AD and FTD may require different caregiving approaches. For example, caregiving for individuals with AD primarily focuses on memory care due to the progressive memory loss associated with the disease. In contrast, caregiving for individuals with FTD emphasizes behavioral management because of the associated changes in impulsive behaviors [4]. Therefore, the inability to accurately diagnose these two types of dementia may result in a decreased quality of life and a misallocation of resources. Accurately distinguishing between AD and FTD is essential.

Electroencephalogram (EEG) analysis is a significant approach for dementia diagnosis, offering advantages such as low implementation cost and widespread availability in medical facilities. Research on the classification of EEG signals between individuals with AD and healthy elderly controls (HCs) has yielded encouraging findings [2,5,6,7,8,9,10,11]. Similarly, research on the classification of EEG signals between FTD and HCs has shown promising results [2,5,6,7,8]. However, achieving similarly successful results for the classification of EEG signals between individuals with AD and those with FTD has been rare. Recent benchmark studies have achieved classification accuracies of over 95% for AD-HC [9] and over 85% for FTD-HC [6] but below 75% for AD-FTD [6]. The lower classification accuracy of AD-FTD hinders the development of an affordable and widely accessible differential diagnosis for these subtypes in clinical settings. This study suggests that further research should focus on improving EEG classification performance for the differential diagnosis of AD and FTD.

The difficulty in achieving AD-FTD classification using EEG data may be attributed to the relatively poor spatial resolution of the EEG system. This inherent limitation of EEG signals may not hinder the identification of dementia from HCs, as other significant features, such as EEG slowing and frequency ratios, remain available. However, EEG features extracted from whole-brain activities may not be practical for differential diagnosis, as these features can overlap across different subtypes [12,13,14,15]. From this perspective, the importance of analyzing EEG data with high spatial resolution is evident.

Analyzing connectivity across EEG system electrodes can enhance spatial resolution. The Pearson correlation coefficient and magnitude coherence are two approaches used to measure connectivity in EEG data across brain areas and have been employed for AD-HC differentiation, as reviewed by [16]. However, both approaches have limitations, including their focus on linear relationships and dependence on signal amplitude. These limitations may restrict classification performance in detecting complex differences within the specific range of brain network disruptions unique to each dementia subtype.

This study introduces a computational framework suggested by information integration theory (IIT) to address the aforementioned issues. The IIT framework quantifies the brain’s integrated information into specific values, known as PHI values, on the basis of information entropy [17]. Previous research has utilized this framework to analyze EEG data for various applied purposes [11,18,19]. This approach allows the calculated PHI values, based on mutual information, to capture both linear and nonlinear interactions between electrodes while being independent of signal amplitude. Thus, the IIT framework could provide a more sensitive measure of connectivity than the Pearson correlation coefficient and magnitude coherence. Previously, we proposed a methodology to process EEG signals into PHI values for machine-learning models aimed at AD-HC classification [11]. Specifically, we used the EEG signals between each pair of electrodes, combined with age and sex, as features to train the classifiers. That study demonstrated improved performance compared to the baseline of a publicly available dataset [5]. In this study, we posit that the proposed methodology has significant implications for the differential diagnosis of AD and FTD since the information communicated between electrodes can be quantified as features, enhancing spatial resolution for training models.

This study selected electrodes for calculating PHI values based on the most likely locations of brain activity that reflect differences in neurological pathology among the targeted dementia subtypes. We focused on the default mode network (DMN), a large-scale brain network implicated in dementia pathologies [20]. Previous studies have suggested that when in a resting state, individuals with AD exhibit reduced synchronization within the DMN compared to HCs, whereas individuals with FTD exhibit increased synchronization within the DMN compared to HCs [21,22,23]. Based on this, we selected electrode pairs (details provided in the Methods section) under the assumption that specific sets of these pairs could achieve high accuracy in AD-FTD classification.

This study aims to distinguish between AD and FTD using the previously proposed methodology [11]. We posed the following research question: “How can the features for AD-FTD classification be narrowed down?” Initially, fifteen features, including PHI values and demographic variables, were utilized. The detailed objectives include: (1) verifying the differences between contributing electrode pairs in distinguishing AD and FTD from HC; (2) identifying the most significant electrode pairs contributing to AD-FTD classification; and (3) determining whether these pairs contribute more or less to the classifications than demographic variables such as age and sex.

## 2. Methods

### 2.1. Dataset

We used a publicly available dataset from OpenNeuro comprising 88 participants: 36 with AD, 23 with FTD, and 29 HCs [5]. The groups differed in average age, Mini-Mental State Examination scores, and sex (see Table 1 for details).

EEG data were captured from all participants under an eyes-closed resting-state protocol using a Nihon Kohden EEG 2100 clinical device, operating at a sampling rate of 500 Hz with a resolution of 10 µV/mm. The device utilized 19 electrodes arranged according to the 10–20 system at the following positions: Fp1, Fp2, F7, F3, Fz, F4, F8, T3, C3, Cz, C4, T4, T5, P3, Pz, P4, T6, O1, and O2. The recordings averaged 13.50 min for the AD group (range: 5.10–21.30 min, total: 485.50 min), 12.00 min for the FTD group (range: 7.90–16.90 min, total: 276.50 min), and 13.80 min for the HC group (range: 12.50–16.50 min, total: 402.00 min).

The dataset provider preprocessed the raw data using the following steps [5]: (1) filtering the data with a Butterworth bandpass filter (0.50–45.00 Hz) and re-referencing it with two reference electrodes; (2) removing bad data (periods exceeding the maximum acceptable 0.5 s window standard deviation of 17) via the artifact subspace reconstruction routine; and (3) rejecting “eye artifacts” and “jaw artifacts” using the automatic classification routine “ICLabel” in the EEGLAB platform.

### 2.2. Computational Framework

IIT divides a system into two distinct parts: Part A and Part B. The output PHI value refers to the mutual information between the two parts in both directions—considering Part A as the source and Part B as the target (*A→B*), and vice versa (*B→A*). This concept was represented using the following notation:

(1) $$PHI\left(S\right)=MI\left(A^{Hmax};B\right)+MI(B^{Hmax};A),$$

where *MI* (*A^Hmax^*; *B*) is the mutual information for *A→B*, and (*B^Hmax^*; *A*) is the mutual information for *B→A*, *PHI* (*S*) indicates the mutual information between two parts within system *S*.

In our methodology [11], we regarded each pair of electrodes as Part A and Part B of a system. Accordingly, we treated the amplitude values over time from each electrode pair as two separate variables. We calculated the mutual information in both directions and summed them to obtain the PHI value for each electrode pair. The mutual information for *A→B* was calculated by the following steps:

(2) $$H\left(Elec.A^{Hmax}\right)=-\sum_{x_{1}\in {Elec.A}^{Hmax}}p\left(x_{1}\right){log}_{2\;}p\left(x_{1}\right),$$

(3) $$H\left(Elec.B\right)=-\sum_{x_{2}\in Elec.B}p\left(x_{2}\right){log}_{2}p\left(x_{2}\right),$$

(4) $$H\left(Elec.A^{Hmax},Elec.B\right)=-\sum_{x_{1}\in {Elec.A}^{Hmax}}\sum_{x_{2}\in Elec.B}p\left(x_{1},x_{2}\right){log}_{2}p\left(x_{1},x_{2}\right),$$

where *Elec. A^Hmax^* is the set of all binned EEG amplitude values from Part A; *Elec. B* is the set of binned EEG amplitude values from Part B that correspond to values from Part A at the same time stamp; *p* (*x*_1_) is the probability of each binned value in *Elec. A^Hmax^*; *p* (*x*_2_) is the probability of each binned value in *Elec. B*; *p* (*x*_1_, *x*_2_) is the joint probability of amplitude values from the two electrodes occurring together; *H (Elec. A^Hmax^)* is the information entropy of *Elec. A^Hmax^*; *H* (*Elec. B*) is the information entropy of *Elec. B*; and *H* (*Elec. A^Hmax^*, *Elec. B*) is the joint entropy of *Elec. A^Hmax^* and *Elec. B*.

Similarly, the mutual information for *B→A* was calculated by repeating steps (2), (3), and (4), treating Part B as the source (i.e., *Elec. B^Hmax^*) and Part A as the target (i.e., *Elec. A*).

Finally, the PHI value for the electrode pair (A and B) is obtained by:

(5) $$PHI\left(EEG\right)=H\left(Elec.A^{Hmax};Elec.B\right)+H(Elec.B^{Hmax};Elec.A),$$

where *PHI (EEG)* represents the PHI value for the electrode pair composed of Part A and Part B.

### 2.3. Data Processing

To analyze the EEG signals using the computational framework described above, we processed the data as follows: (1) discretizing the amplitudes of the EEG signals by rounding them to intervals of 10 μV, and (2) partitioning the data into 60 s epochs. This method treated each rounded amplitude as a distinct state of neural activity at the respective electrode, allowing us to calculate the probabilities of each state within an epoch by counting the frequency of rounded amplitudes over 60 s. To ensure time efficiency, only the first epoch was used to calculate the PHI value for each electrode pair. Each PHI value was calculated within two minutes via a laptop computer (Alienware x14, 12th Gen Intel (R) Core (TM) i7-12700H 2.30 GHz).

Based on evidence from related research [20,21,22,23], we selected 13 pairs of electrodes within the DMN following the 10–20 system to calculate the PHI values. These pairs include: Fp1-Fp2, Fp2-F4, F3-F4, Fp2-F8, Fp2-T4, F7-F8, T5-P3, T3-P3, O1-O2, T3-T5, T4-T6, T4-P4, and T6-P4.

### 2.4. Machine Learning

The obtained PHI values were used as inputs for machine learning models, with binary classification between groups as outputs. Our previous study indicated that the support vector machine (SVM) model with a sigmoid kernel may offer the best performance, achieving higher classification accuracy compared to other models on the same dataset under identical experimental conditions [11]. For a detailed comparison of models, see Table 2. Based on these results, we also employed the SVM model in the current study.

The model performance was evaluated using a confusion matrix, comprising true positive (TP), false positive (FP), true negative (TN), and false negative (FN). Related metrics included classification accuracy, precision, recall, F1 score, and specificity. The equations below detail the calculation for each metric:

(6) Classification accuracy=(TP+TN)/(TP+TN+FP+FN),

(7) Precision=TP/(TP+FP),

(8) Recall=TP/(TP+FN),

(9) F1 score=2∗(Precision∗Recall)/(Precision+Recall),

(10) Specificity=TN/(TN+FP),

Related research frequently employs the leave-one-out cross-validation (LOOCV) technique for validating models in the classification of EEG signals. This method uses one sample as the test set and the remaining samples as the training set for each iteration. This way provides aggregated results by averaging performance metrics across all iterations. LOOCV ensures greater robustness of the trained model than other cross-validation techniques, as suggested by [2]. Most subsequent studies using the same dataset followed this principle to establish comparability of results with each other [5,6,8,9,10,11]. Accordingly, the current study employed LOOCV as the cross-validation technique.

This study applied permutation feature importance to evaluate the significance of the features. We observed changes in classification accuracy when permuting the PHI values from each feature set, relative to the baseline performance. This method produced a ranked list of electrode pairs according to their contribution to model performance.

### 2.5. Analysis

We calculated the PHI values for each electrode pair across all participants. We then classified the PHI values between groups using the SVM models (sigmoid kernel). Additionally, we employed the LOOCV technique as an evaluation procedure. Finally, we performed feature importance analyses and retrained the model accordingly.

## 3. Results

### 3.1. PHI Values

**Figure 1:**
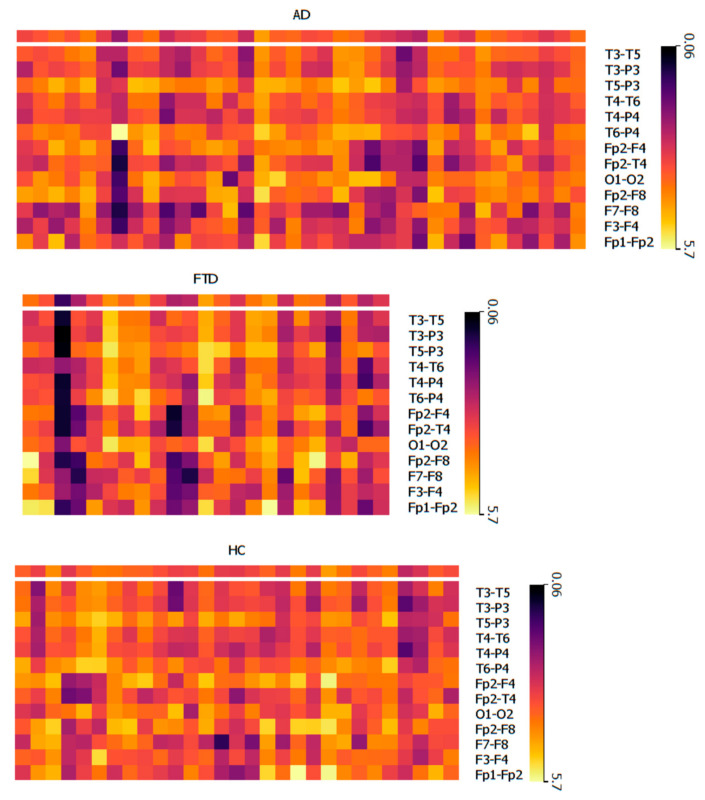
Heatmaps of individual PHI values. The top heatmap represents the AD group, the middle heatmap represents the FTD group, and the bottom heatmap represents the HC group. Each colored rectangle denotes a specific PHI value. Rows correspond to PHI values from the same electrode pair, while columns correspond to PHI values from the same individual. The top row of each heatmap shows the average PHI values for individuals in each group.

Across all electrode pairs, the mean PHI values range from 2.81 to 4.06 for the AD group, 2.78 to 3.92 for the FTD group, and 3.14 to 3.97 for the HC group. The average PHI values for each electrode pair across the AD, FTD, and HC groups were summarized in Table 3. Heatmaps were generated to summarize the PHI values for all participants across the three groups (Figure 1).

### 3.2. Classifications

**Figure 2:**
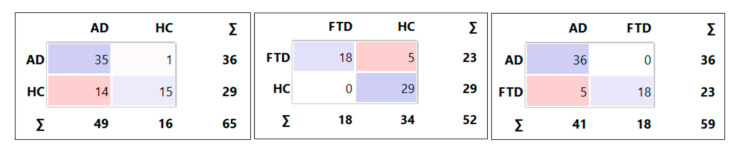
Confusion matrices for AD-HC, FTD-HC, and AD-FTD classifications. The horizontal axis represents the predicted labels, and the vertical axis represents the actual labels.

We used the 13 electrode pairs, along with age (scaled to the range [0, 1] using min-max scaling) and sex, as classification features. The SVM models achieved accuracies of 76.9% for AD-HC classification, 90.4% for FTD-HC classification, and 91.5% for AD-FTD classification. Figure 2 shows the related confusion matrices. For the detailed performance of each model, refer to Table 4.

**Figure 3:**
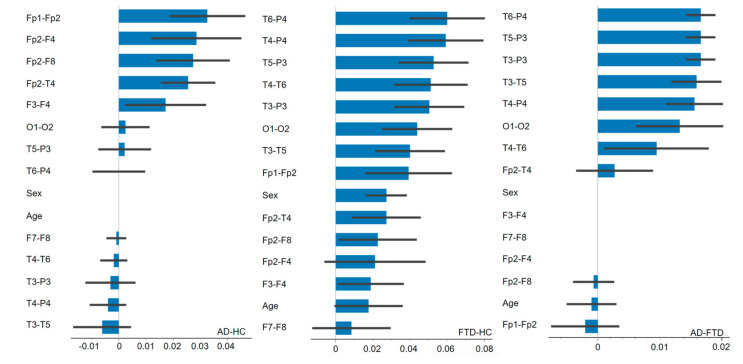
Feature importance analyses for AD-HC, FTD-HC, and AD-FTD classifications using 15 features. The horizontal axis shows a decrease in classification accuracy when a feature is permuted. The vertical axis lists the evaluated features. The blue bars denote the average reduction in classification accuracy for each feature, while the black lines denote the variability in the reduction across multiple permutation iterations.

We performed three feature importance analyses to identify the significant features for each classification, based on the largest decrease in classification accuracy across 50 permutation iterations. The mean decrease in accuracy for the top five features ranges from 0.017 to 0.033 for the AD-HC classification, 0.050 to 0.060 for the FTD-HC classification, and 0.016 to 0.017 for the AD-FTD classification. More details are summarized in Table 5 and Figure 3.

We refined the feature set for AD-FTD classification based on feature importance analysis to improve performance. The SVM model with eight significant features (T6-P4, T5-P3, T3-P3, T3-T5, T4-P4, O1-O2, T4-T6, and Fp2-T4; see Figure 3 under “AD-FTD”) achieved an accuracy of 96.6% (detailed performance in Table 4). This result suggests improved performance than the previous AD-FTD classification with 15 initial features (13 electrode pairs, age, and sex).

## 4. Discussion

This study employed SVM models to distinguish FTD from AD by quantifying EEG data to measure connectivity between electrodes. Thirteen electrode pairs within the DMN and two demographic variables were selected to train the classifiers, achieving a classification accuracy of 91.5%. On the basis of the feature importance analysis, we narrowed the feature set to eight electrode pairs and improved the classification accuracy to 96.6%. This performance surpassed related work on the same dataset, as summarized in Table 6. The following paragraphs interpret the observations.

First, we observed distinct sets of electrode pairs for the AD-HC and FTD-HC classifications. This difference between the two feature sets is justified. In AD, the primary symptom is related to executive function, which is underpinned by abnormal connectivity within the frontal lobe [24]. In contrast, abnormal connectivity across the temporal and parietal lobes impairs language ability, which is one of the primary symptoms of FTD [22,25,26]. The results of the feature importance analysis align with clinical observations regarding the primary symptoms of both dementia subtypes, suggesting the validity of the trained models as a diagnostic approach.

Furthermore, we observed that the AD-FTD classification primarily involves connectivity in the temporal and parietal lobes. This observation aligns with the distinct symptomatic profiles of these two dementia subtypes. As discussed above, the temporal and parietal regions are more directly involved in language and sensory processing. These regions are often more distinctly impaired in FTD than the memory-focused impairments in AD. However, this interpretation raises a further question: “Why do the brain regions underlying the symptoms of FTD, rather than those underlying the symptoms of AD, contribute the most to classification performance?” We argue that the brain regions associated with AD symptoms, unlike those associated with FTD symptoms, are less effective for precise classification because these symptoms (e.g., memory impairments) overlap more with normal aging, making it harder to isolate AD-specific changes among the subtypes. In contrast, the brain regions associated with FTD exhibit more distinct impairments (e.g., language and behavior impairments) than those associated with normal aging or other dementias, facilitating a more accurate differential diagnosis.

The critical point is that the AD-FTD classification requires fewer electrode pairs than distinguishing FTD from HCs, allowing the model to remain independent of most features involved in communication between the frontal and prefrontal regions. This difference could be attributed to the changes in these regions not providing additional predictive power for AD-FTD classification, as their functionality significantly overlaps in both subtypes. Consequently, these regions add unnecessary complexity without improving classification accuracy. However, these features still contribute to the FTD-HC classification, as they are involved in the neurological pathology of FTD.

Another point to consider is that the classification accuracy of AD-FTD increased from 91.5% to 96.6% when the narrower feature set was used, removing certain unnecessary features as suggested by the feature importance analysis (Figure 3 under “AD-FTD”). This improvement could be attributed to the elimination of unnecessary features from the original set of 15, resulting in a less sparse feature space that allows the model to learn more effectively from the data. A comparison of the AD-HC classifications between the current work (76.9%) and our previous results (86.2%, using nine features, including frontal lobe-based PHI values and demographic information [11]) provides additional support for this perspective. These results suggest that reducing the complexity of the feature set improves the model’s performance.

Finally, we observed that the AD-FTD classification minimally relies on the demographic features of age and sex. We propose that EEG connectivity directly measures the functional interactions between electrodes across brain regions. This direct measurement could capture the unique neural network disruptions associated with each subtype as the primary indicator. Demographic features such as age and sex provide population-level insights but do not account for individual variations in brain connectivity patterns. Therefore, the detailed nature of EEG data should be sufficient to distinguish between AD and FTD without additional demographic information.

Compared to alternative computational approaches, the PHI value calculation used in this study demonstrates an advantage in quantifying connectivity between brain regions using EEG data. The Pearson correlation coefficient and magnitude coherence are also used to indicate connectivity in EEG data across brain regions and have been utilized for HC-AD differentiation [16]. However, both approaches have limitations, including a focus on linear relationships and dependence on signal amplitude. In contrast, the PHI value calculation focuses on mutual information, captures both linear and nonlinear interactions, and, being independent of signal amplitude, provides a more sensitive connectivity measure. This advantage is significant for AD-FTD classification, as it allows the detection of complex differences in a limited range of brain network disruptions unique to each condition (e.g., temporal–parietal communication in the current study), resulting in more accurate classification.

While our methodology is advantageous in the classification between AD and FTD, we acknowledge that the range of diseases it can cover is relatively narrow. The current models cannot identify the stage of AD, whether mild, moderate, or severe. Similarly, they are limited in distinguishing between subtypes of FTD, such as behavioral or semantic variants. More importantly, these models are unable to detect mild cognitive impairments (MCI) from dementia or distinguish between MCI subtypes, such as amnestic MCI and non-amnestic MCI. Further investigations in these areas are anticipated.

The limitations of this study are twofold. First, as applied research, we aim to provide a practical solution for differential diagnosis. This principle necessitates that the current methodology ensures good generalizability of the findings, which remains unaddressed. Second, as previous works suggest using the LOOCV technique to ensure model robustness [2], we followed this criterion to maintain comparability with the benchmark. However, we recognize that this technique might cause high variance in the model estimates, potentially leading to overfitting. Future endeavors will replicate the current methodology using multiple datasets and clinical trials to address these concerns.

## 5. Conclusions

### 5.1. Summary

This study classified FTD and AD using EEG data by quantifying connectivity between electrodes through the calculation of the PHI value based on IIT. We selected 13 electrode pairs and calculated PHI values as features to train an SVM model, achieving a classification accuracy of 91.5%. Feature importance analysis suggested that the top five features for this classification are related to the communication between electrodes in the temporal and parietal regions. We observed that this feature set differs from the main features used for identifying AD, and while it overlaps with those used for identifying FTD, it is more narrowly focused. Additionally, demographic features such as age and sex contribute minimally to the AD-FTD classification. Notably, narrowing the feature set by removing features significant to the AD-HC classification improved the AD-FTD classification accuracy from 91.5% to 96.6%. We concluded that connectivity across electrode pairs unique to the FTD-HC classification facilitates highly accurate EEG-based AD-FTD classification.

### 5.2. Contribution

The findings of this study enhance the understanding of spatial information in the differential diagnosis of AD and FTD using EEG signals. Previous research has highlighted the significant role of connectivity across brain regions in this differentiation. However, most of these findings are based on group-level analysis rather than individual-level data [27]. Only a few studies have investigated the enhancement of functional connectivity-based features for diagnostic classification, confirming the distinguishing significance of hypoconnectivity in the frontotemporal network among individuals with FTD [28]. The current study revealed that different connectivity patterns involving the parietal lobe, characterized by PHI values, influence individual-level AD-FTD classification. This finding aligns with group-level evidence from related research [29,30], underscoring the role of the parietal lobe in FTD pathology. Our work translates knowledge from group-level differences to individual-level classifications, contributing to the field of differential diagnosis between AD and FTD.

### 5.3. Future Research

The current methodology still has room for improvement in distinguishing between AD and FTD for differential diagnosis. In this study, we calculated PHI values using EEG recordings with a fixed duration of 60 s. However, according to IIT, PHI values can be updated in tens of milliseconds [17]. In the future, we intend to increase the temporal resolution by processing the EEG signal time series using fractal analysis to capture the dynamic nature of PHI values. We anticipate that analyzing the temporal patterns of dynamic PHI values could enhance the classification accuracy between AD and FTD.

## Figures and Tables

**Table 1:** Descriptive statistics for the dataset.

Group	*N*	Males (*N*)	Age (Mean ± SD)	MMSE ^1^ (Mean ± SD)
AD ^2^	36	12	66.39 ± 7.89	17.75 ± 4.50
FTD ^3^	23	14	63.60 ± 8.20	22.17 ± 8.22
HC ^4^	29	18	67.90 ± 5.40	30

^1^ Mini-Mental State Examination, a 30-point questionnaire used to assess cognitive impairment for dementia risk screening. ^2^ The group of participants diagnosed with Alzheimer’s disease. ^3^ The group of participants diagnosed with frontotemporal dementia. ^4^ The group of healthy elderly controls.

**Table 2:** Benchmark of machine learning models for AD-HC classification [11].

Model	Hyperparameter	Classification Accuracy	F1 Score
SVM ^1^	sigmoid kernel	0.860	0.860
MLP ^2^	1 hidden layer, 10 neurons	0.770	0.760
KNN ^3^	k = 21	0.690	0.690

^1^ A support vector machine model. ^2^ A multilayer perceptron model. ^3^ A *k*-nearest neighbors model.

**Table 3:** Descriptive statistics for the PHI values.

Electrode Pair	AD Mean (SD) ^1^	FTD Mean (SD) ^2^	HC Mean (SD) ^3^
Fp1-Fp2	3.25 (0.89)	3.29 (1.44)	3.64 (0.97)
Fp2-F4	3.54 (0.94)	3.17 (1.26)	3.97 (0.84)
F3-F4	2.96 (0.78)	2.97 (0.89)	3.50 (0.65)
Fp2-F8	3.65 (0.95)	3.43 (1.38)	3.92 (0.90)
Fp2-T4	2.99 (0.89)	2.78 (1.10)	3.20 (0.71)
F7-F8	2.81 (1.05)	2.95 (1.20)	3.25 (0.98)
T5-P3	4.06 (0.68)	3.92 (1.11)	3.90 (0.78)
T3-P3	3.18 (0.67)	3.19 (1.10)	3.14 (0.76)
O1-O2	3.81 (0.87)	3.81 (0.87)	3.71 (0.73)
T3-T5	3.44 (0.70)	3.39 (0.99)	3.33 (0.76)
T4-T6	3.35 (0.60)	3.22 (0.94)	3.28 (0.60)
T4-P4	3.11 (0.55)	3.08 (1.10)	3.14 (0.68)
T6-P4	4.04 (0.64)	3.66 (1.07)	3.92 (0.67)

^1^ The mean and standard deviation of PHI values in the AD group. ^2^ The mean and standard deviation of PHI values in the FTD group. ^3^ The mean and standard deviation of PHI values in the HC group.

**Table 4:** Comparison of classification performance among SVM models.

	Features	Train Time (s)	Test Time (s)	Classification Accuracy	F1	Precision	Recall	Specificity
AD-HC ^1^	15	0.376	0.195	0.769	0.754	0.814	0.769	0.720
FTD-HC ^2^	15	0.253	0.162	0.904	0.902	0.918	0.904	0.879
AD-FTD ^3^	15	0.357	0.174	0.915	0.913	0.926	0.915	0.867
AD-FTD	8	0.206	0.089	0.966	0.966	0.968	0.966	0.947

^1^ The AD-HC classification. ^2^ The FTD-HC classification. ^3^ The AD-FTD classification.

**Table 5:** Top five features for the AD-HC, FTD-HC, and AD-FTD classifications.

Classification	Feature	Mean Decrease in Accuracy (Mean ± SD)
AD-HC	Fp1-Fp2	0.033 ± 0.014
	Fp2-F4	0.029 ± 0.017
	Fp2-F8	0.027 ± 0.014
	Fp2-T4	0.026 ± 0.010
	F3-F4	0.017 ± 0.015
FTD-HC	T6-P4	0.060 ± 0.020
	T4-P4	0.059 ± 0.020
	T5-P3	0.053 ± 0.019
	T4-T6	0.051 ± 0.020
	T3-P3	0.050 ± 0.019
AD-FTD	T6-P4	0.017 ± 0.002
	T5-P3	0.017 ± 0.002
	T3-P3	0.017 ± 0.002
	T3-T5	0.016 ± 0.004
	T4-P4	0.016 ± 0.004

**Table 6:** Comparison of classifications on the same dataset in the literature.

Study	Comparability ^1^	AD-HC	FTD-HC	AD-FTD
Miltiadous et al. [5] ^2^	YES	0.770 (RF ^3^)	0.731 (MLP)	-
Zheng et al. [6]	YES	0.877 (SVM)	0.827 (SVM)	0.729 (SVM)
Lai et al. [7]	NO	0.910 (KNN)	0.930 (KNN)	0.910 (KNN)
Miltiadous et al. [8]	YES	0.833 (DICE ^4^)	0.750 (DICE)	-
Zheng et al. [9]	YES	0.959 (RF)	-	-
Nedeljković et al. [10]	YES	0.776 (RF)	-	-
Ma et al. [11]	YES	0.862 (SVM)	-	-
Current study 1	YES	0.769 (SVM)	0.904 (SVM)	0.915 (SVM)
Current study 2	YES	-	-	0.966 (SVM)

^1^ This column indicates whether a study’s results are comparable to the baseline for the same classification. A study marked “YES” is comparable to the baseline, as it employed LOOCV for cross-validation (see Section 2.4 Machine Learning for details). A study marked “NO” is not comparable due to differences in the cross-validation methods used. ^2^ This study provided the dataset and the initial baseline results. ^3^ A random forest model. ^4^ A dual-input convolution encoder network model (a novel network architecture).

## Data Availability

The data used in this work were sourced from the OpenNeuro repository at https://openneuro.org/datasets/ds004504/versions/1.0.6 (accessed on 18 August 2023).
